# The association of pre-existing comorbid conditions with COVID-19 severity and post-COVID complications; insights from South Asia

**DOI:** 10.12669/pjms.38.3.5719

**Published:** 2022

**Authors:** Sahrai Saeed, Ronak Rajani

**Affiliations:** 1 Sahrai Saeed, Department of Heart Disease, Haukeland University Hospital, Bergen, Norway; 2 Ronak Rajani, Cardiothoracic Centre, Guy’s & St Thomas’ NHS Foundation Trust, London. School of Biomedical Engineering and Imaging Sciences, King’s College London, United Kingdom

**Keywords:** Coronavirus disease 2019, Cardiovascular complications, Echocardiography, Left ventricular ejection fraction, Strain

Since the Coronavirus disease 2019 (COVID-19) outbreak in December 2019, there has been considerable interest as to how the cardiovascular (CV) system is involved in this condition. In particular, it is now recognised that pre-existing CV risk factors and comorbid conditions are impactful on the severity of COVID-19 disease and that this is truly an unique systemic disease. Hypertension, obesity, diabetes, metabolic syndrome and systemic inflammation, which all are associated with increased risk of target organ damage, weaken the heart’s resistance against the Coronavirus leading to CV complications, particularly in intermediate or high-risk hospitalized patients.^1^,^2^

Common CV complications in COVID-19 are myocardial injury (increased cardiac troponins), myocarditis/myopericarditis, acute coronary syndromes, Takotsubo syndrome, heart failure (both right and left side), pulmonary hypertension, arrhythmias, venous thromboembolism, pulmonary embolism, central venous thrombosis, intracardiac thrombus, stroke and mortality.^1^-^3^ Among these, left (LV) and right ventricular (RV) dysfunction have been widely studied. In the International World Alliance Societies of Echocardiography (WASE) COVID-19 Study, LV dysfunction was found in nearly 20%, and RV dysfunction in 30% of patients with acute SARS-CoV-2 infection.^4^ Age at presentation, previous lung disease, lactic dehydrogenase (LDH), LV longitudinal strain and RV free wall strain were independent predictors of in-hospital mortality. In other studies, the prevalence of cardiac involvement in patients admitted with COVID-19 infection was as high as 36% (elevated troponin concentrations), with troponin being a predictor of death.^5^ However, subclinical cardiac dysfunction in asymptomatic or mild symptomatic patients has not been fully investigated. Recent reports suggest that subclinical myocardial dysfunction (impaired strain) assessed by deformation echocardiography or cardiac magnetic resonance (CMR) imaging is common, not only in patients with moderate or severe disease course, but also in mild cases.^3^

In a recent edition of the *Pakistan Journal of Medical Science (Pak J Med Sci)*, Toori *et al*. presented some interesting data from Pakistan showing that pre-existing comorbid conditions were important risk factors for disease severity and mortality in COVID-19.^6^ This cross-sectional study included 884 reverse transcription polymerase chain reaction (PCR) positive individuals with mean age of 40 years, and 98.5% males. The overall prevalence of comorbid conditions were modest: diabetes 8.6%, hypertension 8.1%, ischemic heart disease (IHD) 2.9% and chronic respiratory disease 2.5%. The prevalence of obesity, an important CV disease risk factor, was not reported, and it was further not clear how hypertension, diabetes and ischemic heart disease were defined. However, it is noteworthy that although all patients were hospitalized, hospitalization *per se* was not medically indicated and only a small proportion of patients (4.8%) had severe disease, while the majority of patients were either asymptomatic (74.8%), or had mild (16.3%) or moderate (4.2%) disease severity.^6^ Perhaps unsurprisingly, patients with no previous CV morbidities had a greater chance of recovery without complications. By contrast, those who died (13/16) had chronic comorbid conditions. These results are consistent with those reports from the American Heart Association COVID-19 Cardiovascular Disease registry demonstrating that CV co-morbidities such as IHD, hypertension, and diabetes dramatically increased the risk of in-hospital mortality in patients with COVID-19 infection.^7^

The large sample size is a major strength of the study of Toori *et al*. Furthermore, the rates of in-hospital CV complications and mortality have been extensively studied in hospitalized patients with moderate or severe COVID-19. The study of Toori *et al*. assessed the rates of in-hospital complications and mortality in a cohort of patients with less severe COVID-19, providing useful insights in these patients. The study was well-designed and the results are interesting and an important contribution to growing body of COVID-19 research. However, we would like to address two important issues which were not covered in the paper of Toori *et al*. Firstly, the authors focused mainly on the importance of pre-existing morbidities with regard to the severity of disease; a typically one-way axis: CV risk factors leading to greater risk of CV complications and death in COVID-19. However, it is now appreciated that COVID-19 *per se* leads to development of new CV morbidities. It includes a bidirectional cause-effect relationship.^2^ It has been increasingly shown that after recovery from COVID-19, patients may exhibit sustained poor glycemic control, impaired renal function, elevated blood pressure and sustained tachycardia.^2^,^8^ The latter can manifest in various forms such as common sinus tachycardia, inappropriate sinus tachycardia or postural orthostatic tachycardia syndrome (POTS).^9^ In addition, exercise intolerance, dyspnea, and other symptoms which are often referred to as long COVID (discussed later) may be evident in some patients. Subclinical myocardial dysfunction by strain imaging, persistent myocardial inflammation and edema by CMR imaging and asymptomatic ECG changes such as bundle branch blocks, interventricular conduction delays and ST-segment and T-wave changes following recovery from COVID-19 in milder cases are not uncommon either. Secondly, long COVID symptoms are common, not only in high-risk hospitalized patients but also in younger home-isolated patients with milder disease. In a prospective study of 312 patients (247 home-isolated and 65 hospitalized) in Western Norway, at six month follow-up, 61% of all patients had persistent symptoms.^10^ The severity of persistent symptoms was independently associated with the severity of COVID-19 disease at baseline. A total of 52% of home-isolated young adults (aged 16-30 years) had symptoms such as loss of taste or smell in 28%, fatigue in 21%, dyspnea in 13%, impaired concentration in 13% and memory problems in 11%. Hence, the population studied by Toori *et al*. require close medium and long term follow-up due to the risk of subclinical myocardial dysfunction and long COVID symptoms, such as long-lasting dyspnea and cognitive impairment. Whether the prevalence and burden of subclinical myocardial dysfunction, post-COVID CV complications and long COVID symptoms differ between South Asian and Western populations, warrant future studies.

## Authors’ Contribution:

**SS** wrote the first draft of the article which was subsequently revised by **RR**.

Both authors approved the final submission.
